# Multi-omics analysis reveals a protective role of endogenous proline in sheep oocyte vitrification and its therapeutic application

**DOI:** 10.3389/fcell.2025.1714529

**Published:** 2025-12-16

**Authors:** Airixiati Dilixiati, Xi Zhao, Aikebaier Aihemaiti, Weijian Li, Chen Fan, Guodong Zhao, Abulizi Wusiman, Xuguang Wang

**Affiliations:** 1 Xinjiang Key Laboratory of Equine Breeding and Exercise Physiology, College of Animal Science, Xinjiang Agricultural University, Urumqi, China; 2 Institute of Microbiology, Xinjiang Academy of Agricultural Sciences, Urumqi, China

**Keywords:** sheep, oocyte, vitrification, multi-omics, metabolic reprogramming, proline, developmental competence

## Abstract

**Introduction:**

Vitrification is a rapid-cooling cryopreservation technique for oocytes and a key method in assisted reproductive technology (ART). During vitrification, oocytes are exposed to high concentrations of cryoprotectants, leading to cryoinjury and osmotic stress that impair oocyte quality and subsequent developmental competence in mammals. However, the complex molecular stress responses evoked by vitrification remain poorly understood.

**Methods:**

Here, we used sheep oocytes to compare metabolome and transcriptome profiles before and after vitrification.

**Results:**

Integrated multi-omics revealed a significant accumulation of the osmoprotectant proline in vitrified oocytes. The upregulation of *PYCR3*, a regulator of proline synthesis, and the downregulation of *P4HA1*, which controls hydroxylation, collectively increased intracellular proline levels through this pathway. Guided by this finding, we supplemented the vitrification medium with 0.5 M L-proline, which significantly improved oocyte quality after warming. L-proline supplementation markedly increased the survival rates of vitrified oocytes, reduced oxidative stress, improved organelle distribution (spindle, endoplasmic reticulum, and mitochondria), decreased mitochondrial ROS levels, increased the mitochondrial membrane potential, mitigated ATP decline induced by cryopreservation, and maintained calcium homeostasis.

**Conclusion:**

These protective effects collectively enhanced the developmental competence of vitrified sheep oocytes.

## 1 Introduction

Oocyte cryopreservation technology represents a revolutionary advancement in reproductive medicine and animal breeding. Vitrification, characterized by rapid cooling, minimal ice-crystal formation, and higher rates of survival, fertilization and pregnancy rates, has progressively replaced conventional slow-freezing methods to become the current mainstream technique (Huang et al., 2021). During vitrification, oocytes are exposed to high concentrations of cryoprotectants, cryoinjury and osmotic stress, which may impair mammalian oocyte quality and subsequent developmental potential (Tharasanit and Thuwanut, 2021). Studies indicate that cryoinjury during freezing induces abnormal intracellular ROS elevation, disrupts calcium homeostasis, triggers oxidative stress and zona pellucida hardening, and causes abnormal distribution/function of organelles such as mitochondria, the endoplasmic reticulum, and the spindle apparatus (Sun et al., 2023). Although vitrification has been preliminarily applied to preserve sheep oocytes, its efficiency remains lower than that in humans and mice (Sun et al., 2023).

Single-cell mass spectrometry, which applies mass spectrometric analysis to individual cells, enables high-precision, high-throughput analysis of macromolecules (proteins, metabolites) within single cells, thereby revealing their biochemical characteristics and functional states. Recent advancements in this technology (Zhu et al., 2024) have facilitated its rapid adoption in developmental biology, with metabolic dynamic developmental maps constructed during oocyte‒blastocyst transitions in mice (Zhao et al., 2021), goats (Ma et al., 2024), sheep (Pan et al., 2024), and pigs (Gao et al., 2023). However, metabolic reprogramming in post-cryopreservation oocytes remains unexplored, a knowledge gap that not only limits precise technical optimization but also may lead to an incomplete understanding of cryodamage mechanisms. As metabolomics serves as a critical tool for elucidating cellular physiological states, this study integrates metabolic profiling with single-cell transcriptomics of vitrified sheep oocytes to identify cryopreservation-induced metabolic reprogramming. The findings aim to guide the optimization of freezing protocols, enhance post-thaw developmental competence, and address critical gaps in ovine reproductive cryobiology.

Proline, an imino acid with a unique cyclic structure, plays multifaceted protective roles in biological systems. Its molecular properties—high solubility, neutral pH, high osmotic pressure, and low toxicity—make it an ideal natural cryoprotectant candidate (Krishnan et al., 2008; Signorelli et al., 2014). L-proline accumulation has been documented in plants, yeast (Yan et al., 2025), and *Drosophila* (Koštál et al., 2016) under various environmental stresses, including freezing temperatures, increasing freeze tolerance. Proline stabilizes protein monomers during cold stress through molecular interaction networks, specifically by suppressing conformational abnormalities and promoting functional recovery (Dotsenko et al., 2019). Its mechanism also involves regulating the hydration microenvironments of macromolecular complexes, notably enhancing cryotolerance by stabilizing the solvent layers of nucleoprotein complexes and membrane structures (Patriarca et al., 2021). The cryoprotective efficacy of L-proline has been validated across species: in sperm cryopreservation, supplementation with 2 mM L-proline improved post-thaw motility, plasma/acrosomal membrane integrity, and antioxidant capacity and reduced *Caspase-3/-9* levels in goat sperm while increasing proline dehydrogenase 1 (PRODH) protein expression (Zhang et al., 2022). Similar benefits were observed in human (Moradi et al., 2022), donkey (Li et al., 2021), and cynomolgus monkey (Li et al., 2003) sperm. In oocyte cryopreservation, Zhang et al. (2016) demonstrated that replacing Ethylene Glycol (E.G.,)/dimethyl sulfoxide (DMSO) with 2 mol/L L-proline increased post-thaw survival rates and reduced intracellular ROS in mouse oocytes, with spindle/chromosome alignment comparable to that of fresh controls. Embryo transfer outcomes, including live birth rates and postnatal body weights, were not significantly different from those of fresh oocyte-derived embryos.

## 2 Materials and methods

### 2.1 Reagents

Unless otherwise specified, all reagents and chemicals were purchased from Sigma‒Aldrich (St. Louis, MO, United States).

### 2.2 Ovine ovary collection and oocyte retrieval

Ovine ovaries were collected from the Xinhualing Sheep Slaughterhouse (Urumqi, China) and immediately placed in thermos containers containing prewarmed (32 °C) physiological saline supplemented with 1% penicillin-streptomycin (BBI Life Sciences, #A600135 and #A610494). The ovaries were transported to the laboratory within 2 h. Upon arrival, the ovaries were rinsed repeatedly with physiological saline until the saline remained clear and then transferred to a 35 °C water bath for stabilization. A 10 mL syringe preloaded with 2 mL of oocyte-washing medium (TCM199-HEPES buffer, Gibco, #12340030) containing 1 mg/mL heparin sodium (Sigma, #H3149), 10% (v/v) bovine calf serum (FBS, Gibco, #16030074), and 0.1% (v/v) penicillin–streptomycin was used to aspirate cumulus–oocyte complexes (COCs) from 3 to 8 mm follicles on the ovarian surface. The aspirated COCs were expelled into a preequilibrated 90 mm culture dish containing washing medium. COCs with 2–3 compact cumulus cell layers and homogeneous cytoplasm were selected under a stereomicroscope on a heated stage (38.5 °C) for subsequent experiments.

### 2.3 *In vitro* maturation (IVM) of oocytes

COCs were rinsed three times in a maturation medium composed of TCM-199 (Gibco, #11150059) supplemented with 10% fetal bovine serum(FBS,Gibco,#F8318), 3 IU/mL follicle-stimulating hormone (FSH Solarbio, #F8470), 1 IU/mL luteinizing hormone (LH Solarbio, #L8040), 1.5 μg/mL β-Estradiol (E_2_ Sigma, #E2758), 0.6 mM sodium pyruvate (Sigma, #P4562), 100 μM L-cysteine (L-cy Sigma, #C7352), and 10 μg/L epidermal growth factor (EGF Sigma, #E4127). The COCs were then transferred into 35-mm Petri dishes containing droplets of the same medium under mineral oil (Sigma, #M5310). All dishes had been pre-equilibrated in a humidified atmosphere of 5% CO_2_ at 38.5 °C for at least 2 h. Following a 22-h culture period under these conditions, cumulus cells were removed by gentle pipetting with 0.1% hyaluronidase (Sigma, #H4272). The denuded oocytes were thoroughly washed five times in an oocyte-handling medium. Only fully denuded metaphase II (MII) oocytes that had extruded the first polar body and exhibited homogeneous cytoplasm were selected for subsequent vitrification and downstream experiments.

### 2.4 Vitrification and thawing of oocytes

The vitrification and thawing procedure was based on and modified from the protocol described by Zhao et al. (2024), utilizing the open-pulled straw (OPS) method for vitrification. During vitrification, MII oocytes are washed three times in basal medium (BM; TCM199-HEPES supplemented with 20% FBS) and then transferred to an equilibration solution [7.5% E.G., (Sigma, #324558) and 7.5% DMSO (Sigma, #D2650)] for 5 min. Subsequently, five oocytes are placed in a vitrification solution [15% E.G., and 15% DMSO in 0.5 mol/L sucrose (Sigma, #S1888)] for 25 s. The oocytes, along with a small amount of cryoprotectant, are drawn into the OPS straw tip and rapidly plunged into liquid nitrogen for storage. After at least 1 week of storage, the oocytes were thawed. For thawing, the BM and thawing solutions I, II, and III are preheated on a warming block. After removing the OPS straw from liquid nitrogen, the tip is quickly dipped into thawing solution I to expel the COCs, which are then transferred sequentially to thawing solutions II and III, with equilibration times of 1 min, 3 min, and 5 min, respectively. Finally, the oocytes were washed three times with BM. All the solutions were maintained at 38.5 °C, and the oocytes were incubated for 1 h in an incubator.

### 2.5 *In vitro* fertilization (IVF) and embryo culture (IVC)

The *in vitro* fertilization (IVF) protocol was adapted from the method described by Li et al. (2024). MII oocytes, thawed or selected, were washed three times in IVF solution and then transferred to a droplet for fertilization. Epididymises from slaughterhouse rams were rinsed three times in saline, cut into pieces in a 90 mm dish, and covered with 2 mL of IVF solution. After 5 min of incubation, free sperm were collected, centrifuged at 1800 rpm for two 5-min cycles, and resuspended to 1 × 10^7^/mL. The sperm were added to a droplet of IVF solution with oocytes. After 16 h of incubation, the oocytes were washed three times in embryo culture medium [with essential and nonessential amino acids (Sigma, #M5550 and #M7145), 4 mg/mL BSA (Sigma, #A9418), and 1 mmol/L glutamine (Sigma, #G8540)] and cultured for 24 h to check the cleavage rate and for 7 days (168 h) to observe the blastocysts and calculate the blastocyst rate.

### 2.6 Metabolomic sample collection and analysis

Oocyte sample collection and metabolomic analysis were performed based on methods from Zhao et al. (2021) and Pan et al. (2024) with optimizations. For each experimental group, three biological replicates were prepared, with each replicate comprising a pool of 50 oocytes. Oocytes were washed five times in 0.9% NaCl (Sigma, #71380) to remove mineral oil. They were then transferred to the side of a 1.5 mL microcentrifuge tube (giving a final volume of 20 μL) using a handheld Pasteur pipette. Approximately 80 μL of 80% methanol (Sigma, #1.06035) precooled on dry ice was immediately added. After collection, the tubes were stored at −80 °C. For analysis, samples were retrieved from −80 °C storage. Samples were centrifuged at 14,000 × g for 15 min at 4 °C, and supernatants were transferred to precooled tubes and dried using a vacuum concentrator. The dried metabolites were resuspended in 30 μL of water containing 0.03% formic acid. After vortexing, the mixture was centrifuged again at 14,000 × g for 15 min at 4 °C, and the supernatant was collected for LC‒MS/MS analysis.

LC‒MS/MS conditions: An ultrahigh-performance liquid chromatography system (Nexera X2 LC-30A, Shimadzu) with an ACQUITY UPLC HSS-T3 column (150 × 2.1 mm, 1.8 μm, Waters) was used. The gradient elution program was as follows: 0–3 min, mobile phase B at 1%; 3–15 min, B increased from 1% to 99%; 15–17 min, B at 99%; 17–17.1 min, B decreased to 1%; and 17.1–20 min, B at 1%. Mobile phase A was a water solution with 0.03% formic acid, and mobile phase B was acetonitrile (ACN) with 0.03% formic acid. The flow rate was 0.25 mL/min, the column temperature was 35 °C, the autosampler temperature was 4 °C, and the injection volume was 20 μL. For MS, a triple quadrupole mass spectrometer (QTRAP 6500+, SCIEX) was used in MRM mode to monitor 111 metabolites (77 ion pairs in positive ion mode and 34 in negative ion mode). The identification method involved injecting standards to optimize MRM parameters and determine retention times. The final method covered key pathways, such as energy, carbohydrate, amino acid, and nucleotide metabolism, and included 111 metabolites. The data were analyzed via MultiQuant software (v.3.0, SCIEX) to review chromatograms and integrate peak areas.

Metabolite peak areas were normalized to the total ion current (TIC) of each sample, rather than to cell number, to reflect the relative abundance of each metabolite within the total detected metabolic content. This normalization approach was adopted because the two oocyte groups exhibited comparable cell sizes and total biomass. Chromatographic data review and peak integration were performed using MultiQuant software (v3.0, SCIEX). Normalized peak areas were used as input variables for subsequent multivariate and univariate statistical analyses, following established protocols described previously (Zhao et al., 2021; Pan et al., 2024). All metabolomic data processing and visualization were conducted using the online platforms MetaboAnalyst (https://www.metaboanalyst.ca) and SRplot (http://www.bioinformatics.com.cn/). Differentially abundant metabolites were identified based on the following combined thresholds: fold change (FC) >1.2 or <0.833, variable importance in projection (VIP) score >1, and *p*-value <0.1. KEGG pathway enrichment analysis of these differential metabolites was subsequently performed using SRplot (v2.0), employing Fisher’s exact test with Benjamini–Hochberg false discovery rate (FDR) correction. Pathways with an FDR <0.05 were considered significantly enriched.

### 2.7 Sample collection and analysis via transcriptomics

Sample Preparation: For each experimental group, three biological replicates were prepared, with each replicate comprising a pool of 10 oocytes. Fresh and frozen oocytes were directly lysed in a buffer containing an RNase inhibitor and stored at −80 °C.

Library Construction and Sequencing: Sequencing libraries were prepared using the SMART-Seq v4 Ultra Low Input RNA Kit for full-length cDNA synthesis and amplification, and the Nextera XT DNA Library Prep Kit (Illumina) for subsequent library construction, following the manufacturers’ instructions. Library quality was verified based on fragment size (350–700 bp) using an Agilent 2100 Bioanalyzer. Paired-end sequencing (2 × 150 bp) was carried out on an Illumina NovaSeq 6000 system.

Data Analysis: After quality control, the paired-end clean reads were mapped to the reference genome using HISAT2 (v2.0.5). DESeq2 was employed to identify differentially expressed genes between the treatment and reference groups, with significance thresholds set at |log2FC| ≥ 1(Fresh vs. Vit), and *p*-value <0.05. GO and KEGG enrichment analyses of the DEGs were performed using the online SRplot platform (http://www.bioinformatics.com.cn/, v2.0) with Fisher’s exact test and Benjamini–Hochberg FDR correction. Background gene set consisted of all genes detected in this study; terms/pathways with FDR <0.05 were considered significant.

### 2.8 Translation of RNA and real-time quantitative PCR

Total RNA (70 oocytes per replicate, n = 3 replicates per group) was reverse-transcribed with the One-Step cDNA Synthesis Kit (TransGen, #AC301-01) according to the manufacturer’s instructions. Briefly, oocytes were washed three times in PBS, transferred into a 0.2 mL tube containing 5 μL of PBS, lysed with 5 μL of the provided lysis buffer, and incubated on ice for 10 min cDNA synthesis was then performed according to the manufacturer’s protocol.

Quantitative real-time PCR (RT-qPCR) was carried out with the SuperReal PreMix Plus kit (TIANGEN, #FP205) following the manufacturer’s specifications. Each 20 μL reaction contained 2 μL cDNA template, 10 μL 2× SuperReal PreMix Plus, 0.6 μL each of forward and reverse primers (10 μmol L^-1^), and RNase-free water to 20 μL. Reactions were run in triplicate on a ViiA 7 Real-Time PCR System (Applied Biosystems) using the following thermal protocol: 95 °C for 15 min; 40 cycles of 95 °C for 10 s and 60 °C for 32 s. Primer sequences and amplification efficiencies are listed in Table 1. Relative mRNA levels were calculated with the 2^(–ΔΔCt)^ method, using β-actin as the reference gene.

**TABLE 1:** Primer information.

Gene	Primer sequences (5′-3′)		Anneal temperature (°C)
	Forward	Reverse	
*β-actin*	GGTTGTCTCCTGCGACTTCA	CAGGGCCTTGAGGATGGAAA	60
*NDUFB10*	TGCCCAACCCTATCACCTAC	CCGCTCAATAAACTCTCTCACG	60
*DYNLRB2*	TTCCCATCCGAACGACCTTG	TTCTGGGGGTCAATGTCACG	60
*LGALS1*	GTAACAGCAAGGACGGTGGG	GAAGGAGACGCATACCTCCG	60
*GABARAPL2*	TCCCACAGTCCAGCCTAACT	GAAGCCGAAAGTGTTCTCGC	60
*LAMTOR2*	AGACCGTTGGCTTCGGAATG	TGATGCTGCTACTTGGGTGAG	60
*MRPS31*	GCCACCCAATAGAGGACAACT	GGGTTCAGGGACTTGCTCTT	60
*HERPUD2*	AACGAACCCTTCCACAAGCA	GCTGGTTGTCTACATTTCCTTGC	60
*LSM7*	ATCGAGTACATGCGAGACCCC	GCAGATGAGCACCACCGAAG	60

### 2.9 Determination of intracellular ROS and GSH levels

Intracellular reactive oxygen species (ROS) and glutathione (GSH) levels were assessed in denuded oocytes using the fluorescent probes H_2_DCFDA and CellTracker Blue, respectively. Oocytes were incubated in 20 μmol/L H_2_DCFDA (Invitrogen, #D399) or 20 μmol/L CellTracker Blue (Invitrogen, #C12881) at 38.5 °C under 5% CO_2_ for 30 min. After thorough washing with PBS, fluorescence images were captured using an inverted fluorescence microscope equipped with FITC and DAPI filters for ROS and GSH detection, respectively. The mean fluorescence intensity per oocyte was quantified with ImageJ software (version 1.54).

### 2.10 Immunofluorescence staining and microscopy

Mature oocytes were fixed with 4% (w/v) paraformaldehyde for 45 min at room temperature (RT), permeabilized with 0.5% Triton X-100 (Solarbio, #T8200) for 1 h at RT, and then blocked with 3% bovine serum albumin (BSA) for 1 h at RT to prevent nonspecific binding. Subsequently, oocytes were incubated overnight at 4 °C with a FITC-conjugated anti-α-tubulin antibody (Sigma, #F2168; 1:2000 dilution). After primary antibody incubation, oocytes were washed three times (5 min per wash) in PBS containing 0.1% Tween-20 (PBST). Finally, cell nuclei were counterstained with 4′,6-diamidino-2-phenylindole (DAPI; Vector Laboratories, #H-1200) for 6 min to visualize DNA. The stained oocytes were mounted and imaged using a laser-scanning confocal microscope (Nikon ECLIPSE Ji).

### 2.11 Staining of ER, mitochondria and mitochondrial ROS in oocytes

To assess the distribution of the endoplasmic reticulum (ER), oocytes were stained with 5 μM ER-Tracker Red (Beyotime, #C1041S) in culture medium at 38.5 °C under 5% CO_2_ for 30 min. After washing, the oocytes were imaged using a confocal microscope. The ER distribution patterns were classified as normal or abnormal based on established morphological criteria (Xu et al., 2019). A normal distribution was characterized by a fine, homogeneous granular pattern throughout the cytoplasm, whereas an abnormal distribution was identified by the presence of one or more large, irregular, and dense clusters.

To assess mitochondrial distribution, oocytes were incubated with 50 nM MitoTracker Green FM (Invitrogen, #M7514) in culture medium at 38.5 °C under 5% CO_2_ for 30 min. After thorough washing with PBS, the oocytes were imaged using a confocal laser scanning microscope. Based on established morphological criteria (Zhao et al., 2024), mitochondrial distribution patterns were classified as either normal (evenly dispersed throughout the cytoplasm) or abnormal (clustered into large aggregates).

Mitochondrial superoxide levels were detected using MitoSOX Red (Invitrogen, #M36008). Oocytes were incubated with 5 μM MitoSOX Red in oocyte culture medium at 38.5 °C under 5% CO_2_ for 20 min. After three washes in pre-warmed medium, oocytes were imaged using an inverted fluorescence microscope. The mean fluorescence intensity was quantified using ImageJ software (version 1.54) after background subtraction.

### 2.12 Staining for ATP content and mitochondrial membrane potential in oocytes

The relative ATP content in oocytes was measured based on established protocols using the BODIPY FL ATP fluorescent probe (Invitrogen, #A12410) (Yin et al., 2021). This method provides a comparative measure of ATP-dependent fluorescence in fixed cells. Briefly, denuded oocytes were fixed in 4% paraformaldehyde for 1 h at room temperature. After fixation and washing, oocytes were incubated with 0.5 µM BODIPY FL ATP for 1 h in the dark. Following three washes in DPBS, oocytes were mounted and imaged using an epifluorescence microscope (Nikon TE2000-S). The fluorescence intensity of each oocyte was quantified using ImageJ software after background subtraction.

Mitochondrial Membrane Potential (ΔΨm) was assessed using a JC-1 assay kit according to the manufacturer’s instructions (Solarbio, #CA1310). Oocytes were incubated with the 10 mM JC-1 staining solution—a concentration validated for use in our oocyte model—at 38.5 °C under 5% CO_2_ for 25 min. After washing, imaging was performed using an inverted fluorescence microscope with consistent acquisition settings (e.g., laser power and exposure time) across all groups. The ΔΨm was calculated as the ratio of red (J-aggregates) to green (J-monomers) fluorescence intensity after quantification with ImageJ.

### 2.13 Measurement of subcellular Ca^2+^ levels

Subcellular Ca^2+^ concentrations were assessed using specific fluorescent indicators. To facilitate dye loading, the zona pellucida of oocytes was first removed by brief treatment with 0.5% pronase. The denuded oocytes were then incubated in M2 medium containing Fluo-4 a.m. (for cytosolic Ca^2+^, [Ca^2+^]_c_), Rhod-2 a.m. (for mitochondrial Ca^2+^, [Ca^2+^]_m_), or Mag-Fluo-4 a.m. (for endoplasmic reticulum Ca^2+^, [Ca^2+^]_ER_) (all from Invitrogen, #F1241,R1244,M14206) at 37 °C under 5% CO_2_ for 20 min in the dark. Following incubation, oocytes were thoroughly washed three times with fresh M2 medium. Imaging was performed using a confocal laser scanning microscope with identical settings for all groups to ensure comparability. The mean fluorescence intensity of each oocyte was quantified using ImageJ software by measuring the average pixel intensity within the defined region of interest (ROI).

### 2.14 Statistical analysis

All experiments were independently repeated at least three times, and data are expressed as the mean ± SEM, are denoted in parentheses as (n). The data were analyzed via a paired samples t-test with GraphPad Prism 10 statistical software. *p* < 0.05 was used as the criterion for significant differences.

## 3 Results

### 3.1 Metabolomic analysis of MII-stage sheep oocytes before and after vitrification cryopreservation

**FIGURE 1:**
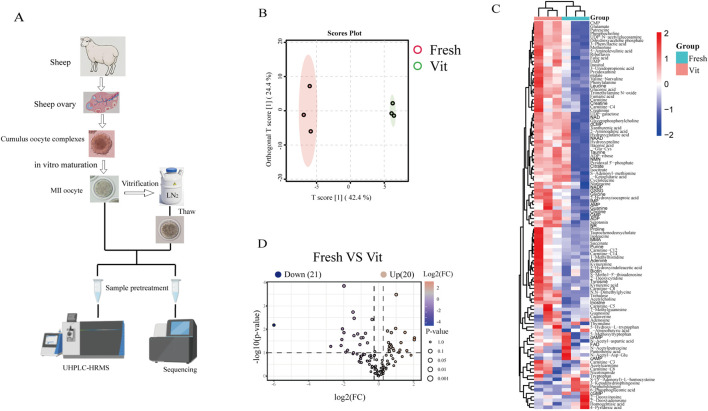
Metabolomic analysis of oocytes before and after cryopreservation. **(A)** Fresh and frozen oocytes were collected for transcriptomic and metabolomic sequencing. **(B)** OPLS-DA score plot of the metabolomics dataset. **(C)** Heatmap visualizing the relative abundance of differentially abundant metabolites in oocytes before and after cryopreservation. **(D)** The volcano plot displays the differentially abundant metabolite levels between the fresh group and the frozen group (downregulated: blue; upregulated: red).

To investigate the physiological impact of vitrification, we characterized the metabolomes of fresh and vitrified oocytes using LC‒MS (Figure 1A). Principal component analysis revealed clear separation between groups, with fresh oocytes clustering tightly on the left and vitrified oocytes forming a distinct group on the right, indicating pronounced cryopreservation-induced metabolic alterations (Figure 1B). Comparative analysis identified 20 significantly upregulated and 21 downregulated metabolites (Figures 1C,D). Several upregulated osmoprotectants, including trehalose, proline, taurine, and inositol, suggested an activated osmotic stress response. We also observed perturbations in nucleotide metabolism, marked by elevated UMP, CMP, and dCMP and decreased dAMP, cAMP, and cGMP. Furthermore, shifts in metabolites related to methylation balance implied epigenetic dysregulation, with upregulated methionine, S-adenosylmethionine (SAM), and 1-methylhistidine, alongside downregulated S-(5′-adenosyl)-L-homocysteine (SAH), choline, and N,N-dimethylglycine.

**FIGURE 2:**
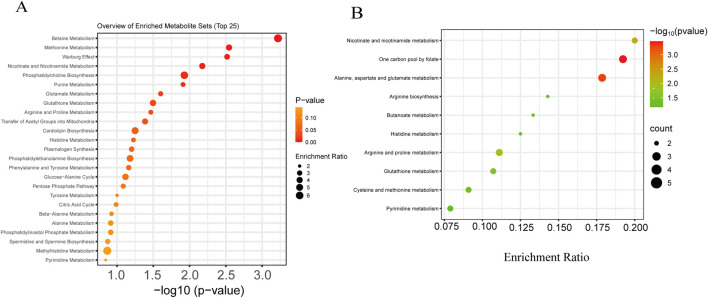
Functional enrichment and pathway analysis of differentially expressed metabolites in oocytes after cryopreservation. **(A)** Functional enrichment analysis of differentially expressed metabolites in oocytes before and after freezing. **(B)** KEGG pathway enrichment analysis of the differentially expressed metabolites in oocytes before and after freezing.

Functional enrichment analysis performed on the MetaboAnalyst platform revealed that the differentially abundant metabolites were significantly enriched in pathways related to lipid and energy metabolism (Figure 2A). The most prominently altered pathways included betaine metabolism, suggesting its critical role in methyl donor homeostasis and cellular osmoregulation; methionine metabolism and the Warburg effect, indicating a potential link between glycolytic reprogramming and amino acid metabolism; and single-carbon pool by folate, alanine, aspartate, and glutamate metabolism, nicotinate and nicotinamide metabolism, phosphatidylcholine biosynthesis, glutathione metabolism, and arginine and proline metabolism.

KEGG pathway enrichment analysis demonstrated that the differential metabolites were most significantly enriched in folate-mediated one-carbon metabolism and alanine, aspartate, and glutamate metabolism (Figure 2B), reflecting profound perturbations in nucleotide synthesis, methylation flux, and amino-acid turnover. The concurrent enrichment of pathways involving nicotinate and nicotinamide, arginine and proline, and glutathione metabolism further indicates that vitrification triggers a global metabolic shift in oocytes, affecting core processes including energy production, redox homeostasis, and amino-acid utilization.

### 3.2 Transcriptomic analysis of oocytes before and after vitrification cryopreservation

**FIGURE 3:**
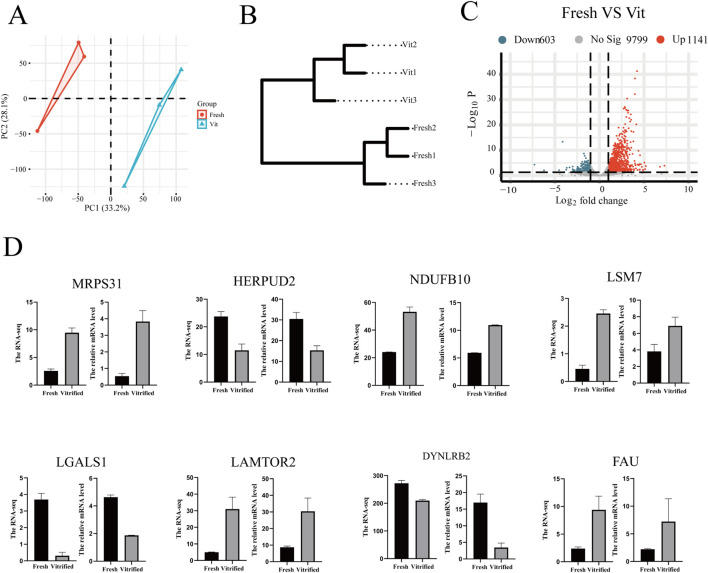
Transcriptomic profiling reveals significant alterations in oocytes following vitrification. **(A)** PCA plot of transcriptomic data demonstrating global gene expression differences between the vitrified and fresh oocyte groups. **(B)** Hierarchical clustering dendrogram of oocyte transcriptome samples (fresh vs. Vit). **(C)** A volcanic plot showing the genes that differ between groups, with red representing upregulated genes and blue representing downregulated genes. **(D)** RT‒qPCR validation of differentially expressed genes in the oocyte transcriptome (fresh vs. vit).

Transcriptomic profiling was performed to investigate the molecular alterations in oocytes induced by vitrification cryopreservation. Principal component analysis (PCA) showed clear separation between fresh and vitrified (Vit) groups, indicating distinct transcriptomic profiles (Figure 3A). This separation was further supported by hierarchical clustering analysis (Figure 3B). Differential expression analysis (|log_2_FC| > 1, *p* ≤ 0.05) identified 1,744 differentially expressed genes (DEGs), including 1,141 upregulated and 603 downregulated genes in vitrified oocytes (Figure 3C). The reliability of the RNA-seq data was confirmed by quantitative real-time PCR (qPCR) validation of a subset of randomly selected DEGs, which showed consistent expression trends (Figure 3D).

**FIGURE 4:**
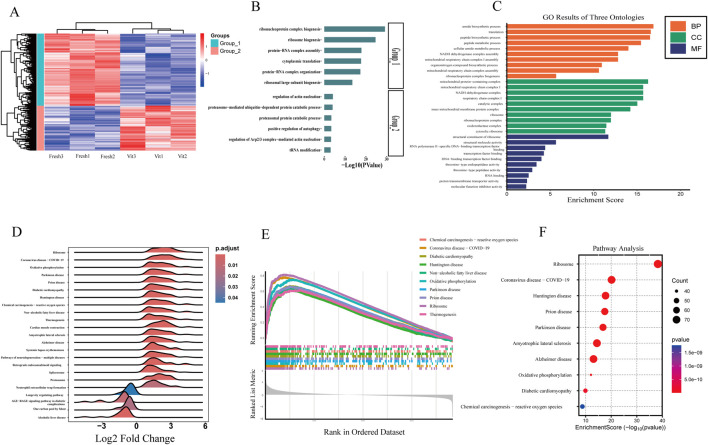
Functional characterization and pathway enrichment of transcriptomic alterations in vitrified oocytes. **(A,B)** Oocyte transcriptome heatmap showing the overall distribution of differentially expressed genes whose expression was segregated into two clusters. **(C)** GO enrichment analysis of differentially expressed genes in the oocyte transcriptome (vitrified vs. fresh). **(D)** GSEA ridge plot. **(E)** GSEA enrichment score fold plot. **(F)** Enrichment analysis of differentially expressed genes in the KEEG pathway in sheep oocytes before and after freezing.

A heatmap of the DEGs revealed two distinct clusters corresponding to fresh and vitrified oocytes (Figures 4A,B). Cluster 1 (fresh oocytes) was characterized by high expression of genes involved in ribonucleoprotein complex biogenesis, protein–RNA complex assembly, and cytoplasmic translation. In contrast, Cluster 2 (vitrified oocytes) was enriched in genes related to actin nucleation regulation, proteasome-mediated ubiquitin-dependent protein catabolism, proteasomal protein degradation, and positive regulation of autophagy. These findings suggest that vitrification disrupts ribosomal function and protein synthesis, enhances proteolysis, impairs actin dynamics (potentially affecting spindle integrity), and activates autophagy.

Gene Ontology (GO) enrichment analysis of the DEGs highlighted several key functional (Figure 4C) categories:Biological processes (BP): “translation,” “amide biosynthetic process,” “mitochondrial respiratory chain complex assembly,” and “organonitrogen compound biosynthetic process,” indicating broad dysregulation of metabolic and mitochondrial activities.

Cellular components (CC): “ribosome,” “mitochondrial protein complex,” “ribonucleoprotein complex,” “mitochondrial inner membrane,” and “oxidoreductase complex,” reflecting alterations in the distribution and organization of key functional complexes.

Molecular functions (MF): “ribosomal structural constituent,” “RNA binding,” and “proton transmembrane transporter activity,” underscoring disruptions in translational machinery, nucleic acid interactions, and transmembrane transport processes.

Gene set enrichment analysis (GSEA) of the transcriptomic data highlighted significant alterations in several key pathways, including ribosome, oxidative phosphorylation, and neurodegenerative diseases such as Parkinson’s and Alzheimer’s (Figures 4D,E). These findings suggest that enhanced energy metabolism—particularly oxidative phosphorylation—may serve as a compensatory mechanism against cryostress, while the suppression of neurodegenerative pathways could reflect active mitigation of misfolded protein aggregation, as exemplified by the downregulation of prion disease-related pathways. Further analysis based on enrichment score rankings identified pronounced activation of chemical carcinogenesis–reactive oxygen species (ROS) and COVID-19-related pathways in vitrified oocytes, indicating the induction of oxidative stress responses and antiviral defense mechanisms. Enrichment of pathways associated with diabetic cardiomyopathy and non-alcoholic fatty liver disease (NAFLD) also suggests potential dysregulation of lipid metabolism and mitochondrial integrity.

Consistent with the GSEA results, KEGG pathway enrichment analysis revealed significant enrichment of pathways related to protein synthesis, energy metabolism, and neurodegenerative diseases (Figure 4F). Among these, the ribosome and oxidative phosphorylation pathways were the most prominently altered, pointing to suppressed ribosomal protein synthesis and remodeled mitochondrial energy metabolism following vitrification. Additionally, pathways involved in chemical carcinogenesis–ROS and multiple neurodegenerative disorders—including Alzheimer’s, Parkinson’s, and Huntington’s diseases—were significantly enriched, further supporting the involvement of oxidative stress and protein homeostasis disruption in post-vitrification oocytes.

### 3.3 Integrated transcriptomic and metabolomic analysis of oocytes before and after vitrification cryopreservation

**FIGURE 5:**
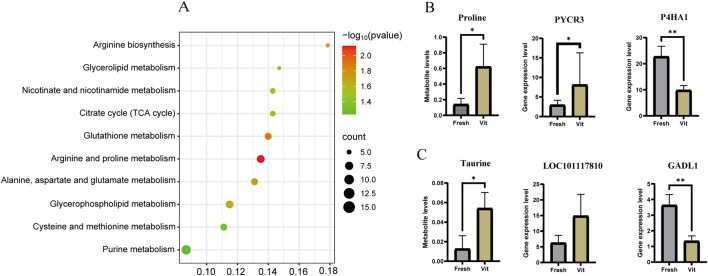
Integrated transcriptomic and metabolomic analysis of oocytes before and after cryopreservation with vitrification. **(A)** Joint analysis of the KEGG pathway enrichment map. **(B)** Bar chart of the expression levels of proline and related pathway genes. **(C)** Bar chart of taurine levels and expression levels of related pathway genes.

KEGG-based co-enrichment analysis of differentially expressed genes (DEGs) and metabolites identified 23 shared pathways, with the top 10 presented in a bubble plot (Figure 5A). Among these, eight pathways were significantly enriched. This included arginine and proline metabolism (–log_10_(p) = 2.00, count = 10) and its subpathway, glutathione metabolism, suggesting the activation of antioxidant systems and nitrogen metabolism in response to cryostress. Core energy pathways such as the TCA cycle (–log_10_(p) = 1.75, count = 8) exhibited coordinated changes with glycerophospholipid metabolism, indicating cellular adaptation through energy reprogramming and membrane lipid remodeling. Significant enrichment of alanine, aspartate, and glutamate metabolism (–log_10_(p) = 1.50, count = 6), along with cysteine and methionine metabolism, further underscored the importance of amino acid metabolism in osmoregulation and methylation processes.

Within the co-enriched arginine and proline metabolism pathway, the upregulation of *PYCR3* (encoding pyrroline-5-carboxylate reductase) and downregulation of *PH4A1* (proline hydroxylase) collectively contributed to elevated intracellular proline levels (Figure 5B). Analysis of taurine-related pathways identified two regulators of L-cysteine conversion: *GADL1*, which was significantly upregulated (log_2_FC = 1.445, *p* < 0.001), and *LOC101117810*, which was downregulated (log_2_FC = −1.23, *p* < 0.05) in vitrified oocytes (Figure 5C).

### 3.4 Identification of L-proline as a candidate additive

Integrated transcriptomic and metabolomic analyses from our preliminary studies identified the arginine and proline metabolism pathway as the most significantly enriched (Table 2), which prompted us to select proline as a candidate additive for the vitrification solution. This decision was based on its dual relevance: first, as a central metabolite in the identified pathway with a known role as an osmolyte in osmotic and cold stress responses; and second, as a well-documented cryoprotective agent in various biological systems. Since L-proline is the exclusively occurring natural stereoisomer, it was incorporated into the vitrification solution for subsequent experimental validation.

**TABLE 2:** Joint pathway analysis of the top five significantly enriched KEGG pathways.

KEGG pathway	Total	Expected	Hits	raw.pval	-LOG10(*p*)
Arginine and proline metabolism	74	4.1994	10	0.0078868	2.1031
Glutathione metabolism	57	3.2347	8	0.013654	1.8647
Arginine biosynthesis	28	1.589	5	0.018683	1.7286
Alanine, aspartate and glutamate metabolism	61	3.4617	8	0.0201	1.6968
Glycerophospholipid metabolism	87	4.9372	10	0.023361	1.6315

### 3.5 Effects of L-p on the survival and subsequent development of vitrified frozen sheep oocytes

### 3.6 Effects of L-proline on oocyte survival and development

This study evaluated the impact of L-proline at various concentrations (0, 0.5, 1, and 2 M) on the vitrification cryopreservation of MII-stage sheep oocytes (Table 3). Key findings included the survival rate. Compared with the control group, the 0.5 M L-proline group presented a significantly greater post-thaw survival rate (98.01% ± 0.79% vs. 86.29% ± 24.74%, *p* < 0.01), indicating optimal cryoprotective efficacy at this concentration. At 2 M L-proline, the survival rates decreased drastically (3.74% ± 6.21% vs. the control, 86.29% ± 24.74%, *p* < 0.01). Cleavage rate: Vitrified oocytes presented lower fertilization rates than fresh controls did (40.50% ± 1.91% vs. 66.42% ± 2.33%, *p* < 0.01). Compared with the control, L-proline supplementation (0.5 M) improved cleavage rates (44.20% ± 1.66% vs. 40.50% ± 1.91%), but the difference was not significant (*p* > 0.05). Compared with those of the 0.5 M group, the cleavage rates of the 1 M L-proline group were significantly lower (37.82% ± 1.17% vs. 44.20% ± 1.66%, *P* < 0.05). The blastocyst rate of vitrified oocytes was markedly lower than that of fresh controls (4.22% ± 1.46% vs. 9.53% ± 1.31%, *p* < 0.01). Compared with the vitrified control group, the 0.5 M L-proline group presented a substantially greater blastocyst rate (43.80% ± 3.10% vs. 4.22% ± 1.46%, *p* < 0.01). The addition of 0.5 M L-proline to the vitrification medium significantly increased the post-thaw survival, cleavage, and blastocyst rates of sheep MII oocytes. This concentration was selected for subsequent experiments.

**TABLE 3:** Effects of L-p on the survival and subsequent development of vitrified frozen sheep oocytes.

L-P concentration	Total number of oocytes (%)	Viable number of oocytes (%)	Survival rate (%)	Cleavage stage embryo count (n)	Cleavage rate (%)	Blastocyst count (n)	Blastocyst rate (%)
Fresh	212			141	66.42 ± 2.33Aa	61	43.80 ± 3.1Aa
0	338	294	86.29 ± 2.47Aa	117	40.50 ± 1.91BCbc	6	4.22 ± 1.46Bb
0.5 M	243	238	98.01 ± 0.79Bb	105	44.20 ± 1.66Bb	10	9.5 ± 1.31Cc
1 M	275	256	93.20 ± 0.98Bb	97	37.82 ± 1.17Cc	3	2.75 ± 1.34Db
2 M	130	5	3.74 ± 6.21Cc	0	0	0	0

Data are presented as the mean ± SEM. Different lowercase and uppercase superscript letters within the same column denote statistically significant differences at *p* < 0.01.

### 3.7 Effects of L-proline on oxidative stress

**FIGURE 6:**
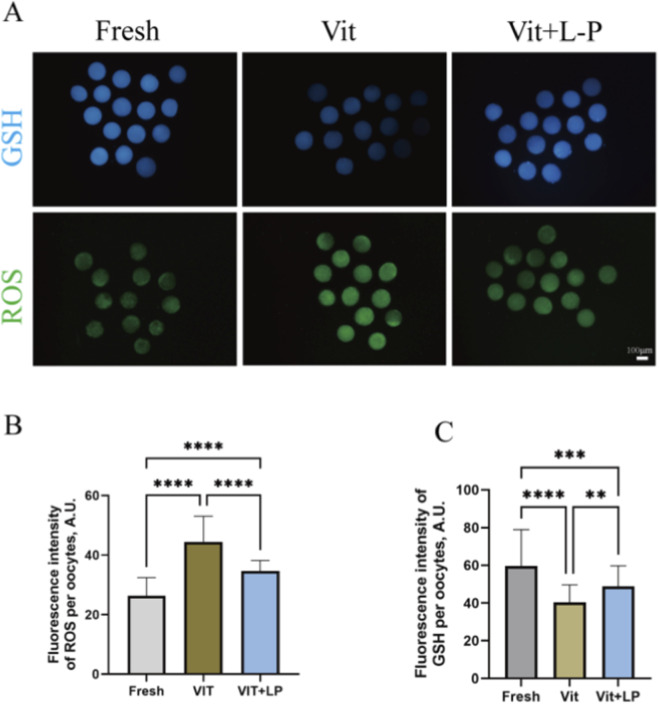
Effects of L-proline on oxidative stress *in vitro*. **(A)** Oocyte levels of GSH (blue) and ROS (green) in oocytes across various groups; scale bar, 50 μm. Bar = 100 µm. **(B)** Relative Fluorescence Intensity of ROS Across Experimental Groups. **(C)** GSH relative fluorescence intensity levels across experimental groups. The data are expressed as the means ± SEMs. **p < 0.01, ***p < 0.001, and ****p < 0.0001.

L-proline can effectively increase post-cryopreservation oocyte survival and developmental capacity. However, cryopreservation may induce oxidative stress, increasing reactive oxygen species (ROS) and potentially causing apoptosis. To explore the impact of L-proline on cryopreservation-induced oxidative stress, this study stained oocytes from different groups with H2DCFDA and Cell Tracker to detect ROS and glutathione (GSH) levels (Figure 6A). The results revealed that the freeze‒thaw group presented significantly higher ROS levels than the fresh group (44.48 ± 1.18, n = 53, Vit; vs. 26.4 ± 0.83, n = 51, Fresh, *p* < 0.01; Figure 6B) and lower GSH levels (40.4 ± 1.21, n = 57, Vit; vs. 59.69 ± 2.75 n = 49, Fresh, *p* < 0.01; Figure 6C), indicating vitrification- and warming-induced oxidative stress. After 0.5 M L-proline treatment, the ROS levels decreased significantly (34.61 ± 0.50, n = 57, Vit + LP; vs. 44.48 ± 1.18, n = 53, Vit, *p* < 0.01; Figure 6B), and the GSH levels increased significantly (48.74 ± 1.42, n = 60, Vit + LP; vs. 40.4 ± 1.21, n = 53, Vit, *p* < 0.01; Figure 6C). In conclusion, L-proline can reduce ROS levels and increase GSH levels in oocytes after cryopreservation, thereby mitigating oxidative stress.

### 3.8 Effects of L-proline on spindle morphology

**FIGURE 7:**
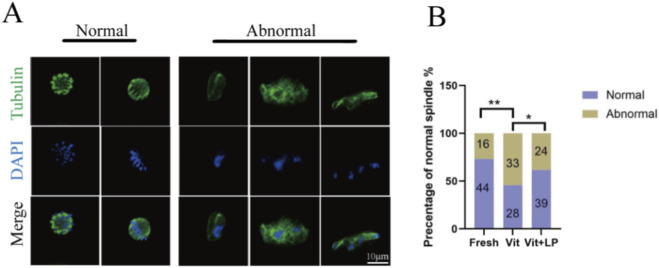
Effect of L-proline on the spindle morphology of vitrified oocytes. **(A)** Immunofluorescence was used to stain mature oocytes for α-tubulin (in green) and chromosomes (in blue); scale bar = 10 µm. **(B)** Proportion of oocytes with normal spindle morphology across experimental groups. The data are expressed as the means ± SEMs. *p < 0.05, **p < 0.01.

Cryopreservation can disrupt oocyte spindle structure, affecting subsequent development. Immunofluorescence staining was used to examine spindle assembly and chromosome alignment in oocytes from different groups. The results (Figures 7A,B) revealed that the number of oocytes with normal spindle structure was significantly lower in the frozen-thaw group than in the fresh group (45.454 ± 3.09, n = 61, Vit; vs. 73.18 ± 5.04, n = 60,Fresh, *p* < 0.01). After treatment with 0.5 M L-proline, the number of oocytes with a normal spindle structure increased significantly (61.84 ± 3.43, n = 63, Vit + LP; vs. 45.454 ± 3.09, n = 61, *p* < 0.05), indicating that L-proline helps preserve spindle integrity in cryopreserved oocytes.

### 3.9 Effects of L-proline on organelle distribution

**FIGURE 8:**
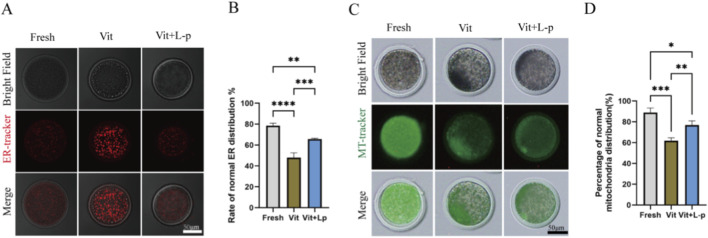
Effects of L-proline on the morphology of the endoplasmic reticulum and mitochondria in vitrified frozen sheep oocytes. **(A)** Endoplasmic Reticulum Distribution in Oocyte Groups. The homogeneous cytoplasmic fluorescence distribution was classified as normal endoplasmic reticulum (ER) patterning (e.g., the fresh group), whereas heterogeneous fluorescence aggregation or cytoplasmic dispersion indicated abnormal ER distribution (e.g., the vitrification group). **(B)** Proportion of oocytes with the correct ER distribution. **(C)** Mitochondrial Distribution Patterns in Oocyte Groups. The homogeneous cytoplasmic fluorescence distribution was defined as normal mitochondrial patterning (e.g., the fresh group), whereas disorganized fluorescent aggregates indicated abnormal mitochondrial distribution (e.g., the vitrification group) **(D)** Rate of corrected mitochondrial distribution in oocytes. The data are expressed as the means ± SEMs. *p < 0.05, **p < 0.01, ***p < 0.001, and ****p < 0.0001.

When oocytes are damaged by cryopreservation and oxidative stress increases, it can affect the distribution of organelles. The proper distribution and coordinated functions of organelles are essential for oocyte development. The endoplasmic reticulum (ER) and mitochondria are crucial in oocytes. Therefore, we examined the effects of L-proline on the distribution of these organelles in vitrified–warmed oocytes. The results for ER distribution (Figures 8A,B) revealed that the frozen-thaw group presented a significantly lower rate of normal ER distribution than the fresh group did (48.05 ± 2.65, n = 74, Vit; vs. 78.51 ± 1.43, n = 74, Fresh, *p* < 0.01). After the addition of 0.5 M L-proline, the proportion of normal ER distribution in oocytes increased significantly (48.05 ± 2.65, n = 74, Vit; vs. 65.8 ± 0.44, n = 79, Vit + LP, *p* < 0.01), indicating that L-proline helps maintain normal ER distribution in cryopreserved oocytes. Mitochondrial distribution is shown in Figures 8C,D The proportion of oocytes with normal mitochondrial distribution was significantly lower in the vitrified group than in the fresh group (61.99 ± 1.54,n = 100,Vit; vs. 88.94 ± 2.51,n = 82,Fresh, *p* < 0.01), indicating impaired mitochondrial patterning after cryopreservation. However, supplementation with 0.5 M L-proline significantly increased this proportion (75.74 ± 2.01, n = 100, Vit; vs. 61.99 ± 1.54, n = 78, Vit + LP, *p* < 0.01), suggesting that proline effectively attenuates vitrification-induced mitochondrial distribution abnormalities.

### 3.10 Effects of L-proline on mitochondrial function

**FIGURE 9:**
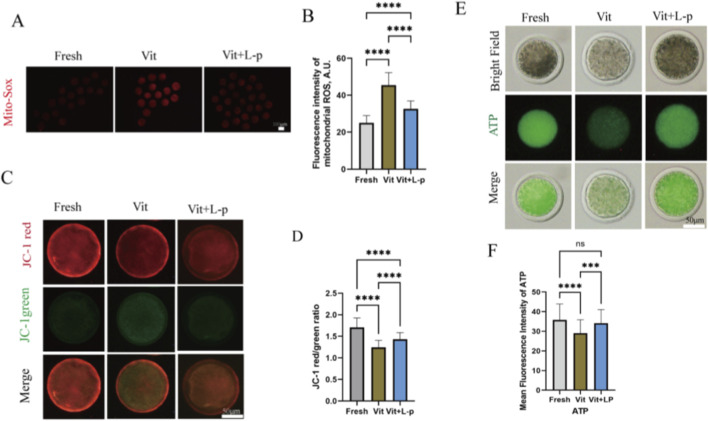
Impact of L-proline on mitochondrial function in vitrified oocytes. **(A)** Mitochondrial superoxide detection via MitoSOX™ Red staining. Higher fluorescence intensity indicates elevated mitochondrial ROS levels. Representative micrographs are shown: fresh, vitrified, and vitrified + L-P. Scale bar: 100 μm. **(B)** Mean fluorescence intensity of MitoSOX™ Red across the experimental groups. **(C)** The CM potential was detected by JC-1 staining. At high mitochondrial membrane potential (ΔΨm), JC-1 accumulates in the mitochondrial matrix and forms J-aggregates that emit red fluorescence (590 nm), indicative of functional mitochondria. Conversely, under low ΔΨm, JC-1 remains monomeric throughout the cytoplasm, emitting green fluorescence (530 nm), indicating mitochondrial depolarization and dysfunction. **(D)** JC-1 fluorescence ratio (red:green) across experimental groups, histogram representing the ratio of red fluorescence (functional mitochondria) to green fluorescence (depolarized mitochondria) in the oocyte groups. **(E)** ATP quantification via the BODIPY™ FL ATP probe. The fluorescence intensity is directly proportional to the ATP concentration. Scale bar: 50 μm. **(F)** Mean fluorescence intensity of BODIPY™ FL ATP across experimental groups. The data are expressed as the means ± SEMs. *p < 0.05, **p < 0.01, ***p < 0.001, and ****p < 0.0001; ns represents no significance.

To further evaluate the functional status of mitochondria, we detected the levels of mitochondrial reactive oxygen species (ROS) using MitoSOX Red fluorescence staining. As shown in Figures 9A,B, vitrified oocytes exhibited a significantly increased mitochondrial ROS level compared to fresh oocytes (45.41 ± 0.81, n = 72, Vit; vs. 25.03 ± 0.47, n = 72, Fresh, *p* < 0.01). Notably, supplementation with 0.5 M L-proline significantly reduced this elevation (32.7 ± 0.50, n = 72, Vit + LP; vs. 45.41 ± 0.81, n = 72, Vit, *p* < 0.01), indicating that L-proline effectively mitigates vitrification-induced mitochondrial ROS production.

The mitochondrial membrane potential (ΔΨm), which is generated by the asymmetric distribution of protons and ions across the inner mitochondrial membrane and is crucial for mitochondrial function, was assessed using JC-1 staining. The JC-1 red/green fluorescence ratio was significantly lower in the vitrified group than in the fresh group (1.24 ± 0.025, n = 42, Vit; vs. 1.708 ± 0.034, n = 42, Fresh, *p* < 0.01), suggesting ΔΨm dissipation(Figures 9C,D). This reduction was effectively rescued in the 0.5 M L-proline supplemented group (1.43 ± 0.023, n = 46, Vit + LP; vs. 1.24 ± 0.025, n = 42, Fresh, *p* < 0.01).

Given that mitochondria are the primary site of ATP production, supplying energy for oocyte competence and subsequent embryonic development, we further measured the intracellular ATP content. Vitrification significantly decreased ATP levels compared to fresh oocytes (28.96 ± 0.835,n = 69, Vit; vs. 35.76 ± 0.9735, n = 69, Fresh, *p* < 0.01,Figures 9E,F). Importantly, L-proline treatment significantly restored the impaired ATP levels (34.11 ± 0.83, n = 69, Vit + LP; vs. 28.96 ± 0.835, n = 69, Vit, *p* < 0.01).

Collectively, these findings demonstrate that L-proline ameliorates vitrification-induced mitochondrial dysfunction in sheep MII oocytes, as evidenced by reduced ROS, restored ΔΨm, and enhanced ATP production.

### 3.11 Effects of L-proline on intracellular Ca^2+^ balance

**FIGURE 10:**
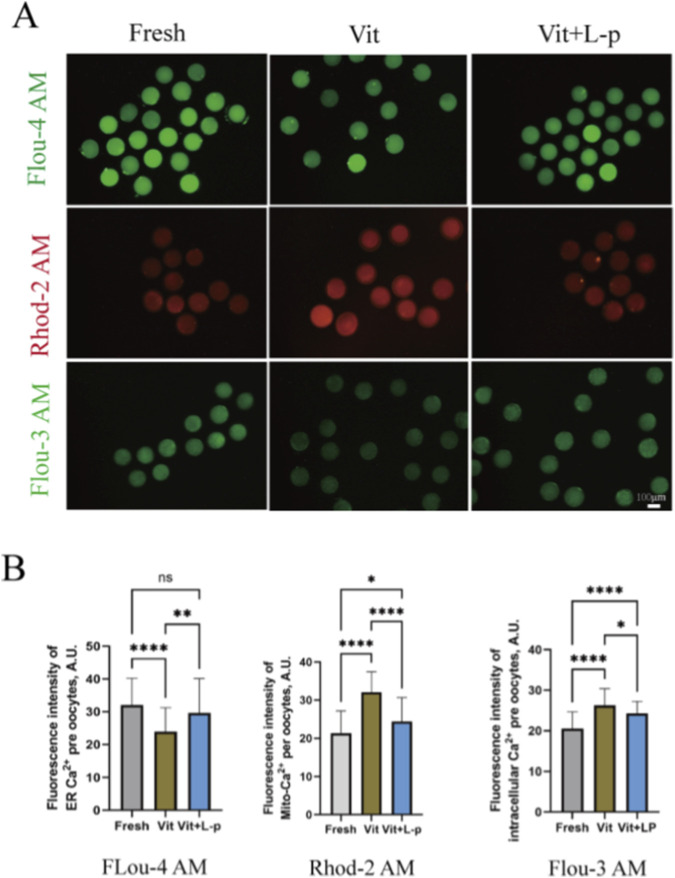
Regulatory effects of L-proline on calcium homeostasis in vitrified Ovine Oocytes. **(A)** Representative Ca^2+^ fluorescence images across experimental groups. **(B)** Quantitative analysis of the mitochondrial Ca^2+^ (Rhod-2 a.m.), endoplasmic reticulum Ca^2+^ (Mag-Fluo-4 a.m.), and cytosolic Ca^2+^ (Fluo-3 a.m.) levels. The data are expressed as the means ± SEMs. *p < 0.05, **p < 0.01, ***p < 0.001, and ****p < 0.0001; ns represents no significance.

Vitrification–warming affects mitochondrial and ER distribution and function. As calcium reservoirs, these organelles are crucial for regulating intracellular Ca^2+^, which is vital for oocyte maturation, fertilization, and early embryonic development. To assess Ca^2+^ levels in these compartments, oocytes were stained with Rhod 2AM ([Ca^2+^]_m_), Mag Fluo-4a.m. ([Ca^2+^]_ER_), and Fluo-3a.m. ([Ca^2+^]_c_) (Figures 10A,B). The results revealed that vitrification and warming significantly reduced [Ca^2+^]_ER_ levels compared with those in the fresh group (32.07 ± 1.18, n = 48, Fresh, vs. 23.91 ± 0.99, n = 55,Vit, *p* < 0.01). L-proline treatment restored the [Ca^2+^]_ER_ levels to near fresh-group levels (29.63 ± 1.55, n = 46,Vit + LP, vs. 32.07 ± 1.18,n = 48,Fresh, *p* > 0.05). The [Ca^2+^]_m_ level increased significantly after cryopreservation (21.36 ± 0.90,n = 41, Fresh, vs. 32.07 ± 0.76, n = 50, Vit, *p* < 0.01), but L-proline treatment reduced it (32.07 ± 0.76, n = 50, Vit, vs. 24.41 ± 0.79, n = 62, Vit + LP, *p* < 0.01). The [Ca^2+^]_c_ level also increased significantly after vitrification and warming (20.51 ± 0.55, n = 57, Fresh, vs. 26.26 ± 0.50, n = 69, Vit, *p* < 0.01), but L-proline alleviated this increase (26.26 ± 0.50, n = 69, Vit, vs. 24.28 ± 0.42, n = 50, Vit + LP, *p* < 0.05). In summary, L-proline can effectively correct cryopreservation-induced intracellular Ca^2+^ imbalances, with significant effects on ER and mitochondrial Ca^2+^ levels.

## 4 Discussion

**FIGURE 11:**
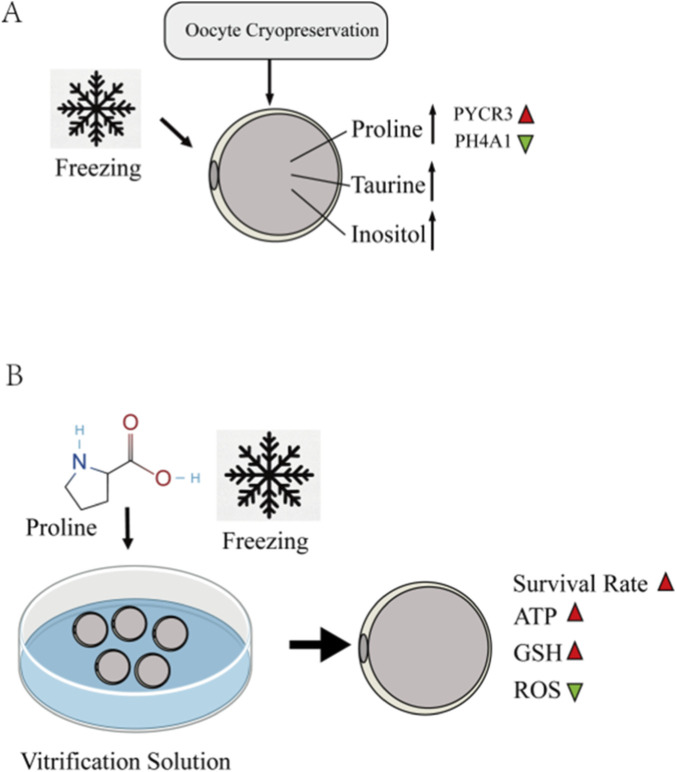
**(A)** After cryopreservation, the endogenous synthesis of osmoprotectants such as proline, taurine, and inositol in ovine oocytes is increased to counteract osmotic stress and low-temperature changes. Supplementation of ovine oocyte freezing medium with 0.5 M L-proline significantly improved the survival rate of cryopreserved oocytes. **(B)** It reduces oxidative stress induced by freezing, ameliorates the distribution of organelles (including the spindle apparatus, endoplasmic reticulum, and mitochondria), lowers mitochondrial reactive oxygen species (ROS) levels, increases the mitochondrial membrane potential, mitigates the decline in ATP levels caused by cryopreservation, and maintains intracellular calcium homeostasis.

In this study, we investigated the transcriptomic and metabolomic alterations in oocytes before and after vitrification, revealing unique mechanisms by which oocytes respond to freezing/hyperosmotic stress. We discovered that post-cryopreservation, oocytes upregulate the synthesis of osmoprotectants—such as proline, taurine, and inositol—to counteract osmotic and temperature changes (Figure 11A). Integrated analysis of both omics approaches highlighted the pivotal role of proline and arginine metabolism during oocyte cryopreservation. Consequently, we identified proline as a potential cryoprotectant additive, which significantly improved the post-thaw oocyte quality and subsequent developmental competence (Figure 11B).

Vitrification improves oocyte survival by employing ultra-rapid cooling to prevent ice crystal formation. However, the low surface-area-to-volume ratio of oocytes restricts dehydration and cryoprotectant permeation, leading to dramatic osmotic fluctuations during freezing and thawing—with osmolarity rising from approximately 300 mOsm/L to 1000–2000 mOsm/L—which induces significant cell volume changes. In response to osmotic stress, cells accumulate organic osmolytes such as glycine and betaine under hyperosmotic conditions, while under hypoosmotic conditions, they release anions and osmolytes through volume-regulated anion channels (VRAC/VSOAC) (Tscherner et al., 2021). Nonetheless, the specific metabolic mechanisms underlying cryotolerance in sheep oocytes remain to be elucidated.

Our study revealed that vitrification led to the upregulation of 20 metabolites and the downregulation of 21 metabolites. Notably, trehalose, proline, taurine, and inositol were significantly accumulated in vitrified oocytes in response to cryopreservation stress. These osmoprotectants help mitigate dehydration-induced damage and macromolecular denaturation by stabilizing proteins and membranes, either through hydrogen bond-mediated vitrification or by acting as compatible solutes. Our findings lend support to the potential of these compounds as cryoprotective additives, which has been empirically demonstrated in previous studies on gamete cryopreservation (Mohammadi et al., 2019; Shiva Shankar Reddy et al., 2010; Zhang et al., 2016; Sanaei et al., 2018).

Targeted metabolomics showed elevated citrate, nicotinamide mononucleotide (NMN) and fumarate. Citrate both fuels the tricarboxylic acid (TCA) cycle and is exported to the cytosol for cleavage into acetyl-CoA, thereby supplying the acetyl donor for histone acetylation and facilitating transcriptional adaptation to energy stress (Mahr et al., 2023). Fumarate accumulation indicates compromised activity of succinate dehydrogenase (SDH) or fumarate hydratase (FH). By succinating and inactivating prolyl hydroxylase P4HA1, fumarate stabilises HIF-1α, which in turn transcriptionally reprogrammes oocytes toward glycolysis to counteract a pseudohypoxic state (Choi et al., 2021). Consequently, oocytes preferentially generate ATP via glycolysis rather than oxidative phosphorylation. Transcriptome-wide KEGG enrichment analysis confirmed significant downregulation of oxidative phosphorylation genes after vitrification. An increased abundance of dihydroxyacetone phosphate (DAP), an intermediate of upper glycolysis, corroborates this switch. Elevated NAD + moreover fuels redox reactions in glycolysis and the TCA cycle, while serving as the obligate co-substrate for SIRT3-mediated deacetylation that bolsters DNA repair, mitochondrial dynamics and mitophagy. These adaptations jointly attenuate ROS accumulation, preserve mitochondrial homeostasis and postpone apoptosis during cryopreservation (Zhang et al., 2024).

Here, we provide the first evidence that mammalian oocytes mount a proline-conserving programme under cold stress by simultaneously up-regulating *PYCR3* (the committed biosynthetic enzyme) and down-regulating *P4HA1* (the key collagen-prolyl hydroxylase that also governs proline catabolism). An analogous “synthesis-on/degradation-off” switch—P5CS induction and PDH repression—underlies the evolutionarily conserved proline surge that protects plants from osmotic shock (Yan et al., 2025). The congruence implies that proline accrual is an ancient, cross-kingdom metabolic defence against low-temperature or hyperosmotic challenge.

Integrative omics pointed to proline metabolism as a node affected by vitrification; we therefore supplemented the freezing medium with 0.5 M L-proline. This simple intervention improved post-thaw survival and subsequent developmental competence of ovine oocytes to the blastocyst stage, echoing earlier observations in the mouse (Zhang et al., 2016). Vitrification inevitably produces transient temperature shifts and nascent ice crystals that evoke oxidative stress, characterised by ROS surge and GSH depletion (Gao and Critser, 2000). Consistent with a protective role, proline-treated oocytes displayed lower ROS intensity and higher total GSH. We speculate that these effects are mediated, at least in part, by proline-dependent provision of cysteine and by transcriptional upregulation of GCLM, the rate-limiting subunit of glutamate–cysteine ligase, thereby accelerating *de-novo* GSH synthesis (Liu et al., 2023).

The meiotic spindle orchestrates accurate chromosome segregation and is exquisitely vulnerable to cryoinjury. Rapid temperature shifts and nascent ice crystals destabilise microtubules, predisposing oocytes to chromosome misalignment and aneuploidy, thereby compromising fertilisation and developmental potential (Bromfield et al., 2009). We demonstrate that supplementing the vitrification medium with proline markedly improves spindle re-assembly and morphology after warming, securing the structural prerequisite for post-thaw embryogenesis. This stabilising effect aligns with the spindle protection previously observed by Zhang et al. (2016) in mouse oocytes, underscoring a conserved benefit of proline in preserving microtubule integrity during cryopreservation.

Mitochondria, the cellular powerhouses, are acutely vulnerable to the thermal and osmotic whiplash of vitrification. Ultrastructural havoc—swelling, fractured cristae and vesiculation—dismantles the filamentous network, depressing ATP output, signalling and apoptosis control (Zhu et al., 2024). We show that 0.5 M L-proline restores ATP content, elevates mitochondrial membrane potential and mitigates mtROS in post-warm oocytes. These functional gains align with cross-species efforts to preserve mitochondrial fitness during oocyte cryopreservation through targeted or non-targeted antioxidants (Shirzeyli et al., 2021; Xiang et al., 2021; Hashimoto et al., 2025; Zhao et al., 2024), underscoring mitochondria as a tractable node for enhancing cryotolerance.

The endoplasmic reticulum (ER) is the hub of protein synthesis and folding; its dysfunction triggers unfolded-protein stress and global cellular decline (Krshnan et al., 2022). ER fragmentation has been documented in cryopreserved oocytes of several species (Barrera et al., 2018; Nottola et al., 2016). Our transcriptomic KEGG analysis revealed enrichment of ER-related pathways—proteasome, RNA degradation and RNA transport—and live-cell imaging showed conspicuous ER dispersion after vitrification. L-proline supplementation restored reticular ER architecture, suggesting a cytoprotective role independent of classic ER chaperones.

ER integrity is functionally coupled to mitochondria via Ca^2+^ signalling; disruption of this coupling compromises oocyte viability (Chen et al., 2024). High concentrations of permeating cryoprotectants (ethylene glycol, DMSO) used during vitrification can perturb Ca^2+^ channels and pumps, leading to cytosolic Ca^2+^ overload. We observed aberrant Ca^2+^ accumulation in warmed oocytes, which was alleviated by proline. Proline is known to form a hydration shell around proteins, minimising denaturation (Fedotova and Dmitrieva, 2016); we therefore speculate that this mechanism preserves the conformation of Ca^2+^-ATPases and store-operated channels, thereby re-establishing Ca^2+^ homeostasis and reinforcing ER–mitochondria crosstalk during cryopreservation.

## Data Availability

The raw sequence data reported in this paper have been deposited in the Genome Sequence Archive (Genomics, Proteomics and Bioinformatics 2025) in National Genomics Data Center (Nucleic Acids Res 2025), China National Center for Bioinformation/Beijing Institute of Genomics, Chinese Academy of Sciences (GSA: CRA033105) that are publicly accessible at https://ngdc.cncb.ac.cn/gsa.
